# Prevalence of Functional Gastrointestinal Disorders and Associated Risk Factors Among Preschool Children in the City of Jeddah and Surrounding Areas: A Cross-Sectional Study

**DOI:** 10.3390/diagnostics15030242

**Published:** 2025-01-21

**Authors:** Mai A. Khatib, Elham A. Aljaaly, Eram Albajri, Nahlaa A. Khalifa, Saleh Khateeb, Sarah M. Ajabnoor, Daniah Radhwan, Khawlah Aljohani, Aisha Y. Hussein

**Affiliations:** 1 Department of Clinical Nutrition, Faculty of Applied Medical Sciences, King Abdulaziz University, Jeddah 21589, Saudi Arabia; ealjaaly@kau.edu.sa (E.A.A.); ealbajri@kau.edu.sa (E.A.); smajabnoor@kau.edu.sa (S.M.A.); dradwan0002@stu.kau.edu.sa (D.R.); kjohani@stu.kau.edu.sa (K.A.); ayousifhussein@stu.kau.edu.sa (A.Y.H.); 2 Food, Nutrition, and Lifestyle Unit, King Fahd Medical Research Center, King Abdulaziz University, Jeddah 21589, Saudi Arabia; 3 Obesity and Lifestyle Unit, King Abdulaziz University Hospital, King Abdulaziz University, Jeddah 21589, Saudi Arabia; 4 Saudi Diabetes Research Group, King Fahd Medical Research Center, King Abdulaziz University, Jeddah 21589, Saudi Arabia; 5 Medical Nutrition Therapy Unit, King Abdulaziz University Hospital, King Abdulaziz University, Jeddah 21589, Saudi Arabia; 6 Department of Respiratory Therapy, Faculty of Medical Rehabilitation Sciences, King Abdulaziz University, Jeddah 21589, Saudi Arabia; nkhalefa@kau.edu.sa; 7 Faculty of Clinical Sciences, Fakeeh College for Medical Sciences, Jeddah 23323, Saudi Arabia; sakhateeb@fcms.edu.sa

**Keywords:** Rome IV Diagnostic Questionnaire, functional gastrointestinal disorders, FGIDs, functional constipation, preschool children, bedtimes, eating pattern

## Abstract

**Background/Objectives:** Functional gastrointestinal disorders (FGIDs) affect children’s daily activities and overall performance due to gastrointestinal symptoms. This study assesses the prevalence and types of FGIDs in children living in Jeddah City and its countryside. It also examines factors that contribute to the incidence of these disorders and their impact on children’s lifestyles. **Methods:** This cross-sectional study was conducted among 285 mothers of preschool children enrolled in kindergartens during the academic year 2020–2021. The Rome IV Diagnostic Questionnaire was sent out online through kindergartens to be filled out by the children’s mothers. The questionnaire assessed the prevalence of FGIDs subjectively through symptoms and their frequency. **Results:** Among the 285 participants, 9% (n = 27) fit the diagnostic criteria for FGIDs. Common FGIDs included functional constipation, 3.5% (n = 10); postprandial distress syndrome, 2.4% (n = 7); functional abdominal pain—not otherwise specified, 1% (n = 3); and functional epigastric pain, 0.7% (n = 2). Significant risk factors for developing FGIDs among the children in the sample included being a preterm baby (*p* < 0.01), being previously diagnosed with a gastrointestinal condition (*p* < 0.010), having a family history of diarrhea or nausea and vomiting (*p* < 0.001 and *p* < 0.01, respectively), skipping lunch at kindergarten (*p* < 0.01), and having pre-existing food allergies (*p* < 0.01). **Conclusions:** FGIDs were prevalent among 9% of children in Jeddah City and its countryside. Functional constipation was the most common disorder. Factors associated with FGIDs in children included preterm birth, being previously diagnosed with a GI condition, a family history of gastrointestinal conditions, irregular eating habits, and food allergies.

## 1. Introduction

Functional gastrointestinal disorders (FGIDs) are a global issue characterized by chronic or recurrent painful gastrointestinal (GI) symptoms [1]. They have a 40% prevalence rate worldwide [2]. In adults, FGIDs include esophageal, biliary, gastroduodenal, bowel, and anorectal disorders, as well as functional abdominal pain syndrome. In children, these disorders are further categorized by age (neonates/toddlers and children/adolescents) and include, but are not limited to, functional bloating, irritable bowel syndrome (IBS), functional diarrhea, and functional constipation [1,3].

FGIDs are not attributed to structural abnormalities or associated with metabolic, systemic, or organic diseases that could explain their symptoms [4,5]. Instead, they are characterized by physiological and morphological disruptions, including visceral hypersensitivity; motility disturbances; altered immune and mucosal function and central nervous system activity; and changes in gut microbiota. These abnormalities tend to co-manifest [6].

FGIDs not only impact the GI system but also the individual’s quality of life and ability to carry out everyday tasks [7]. Some of the commonly reported symptoms found in children with FGIDs are nausea, regurgitation, vomiting, abdominal pain, diarrhea, and constipation [8]. Among the risk factors for developing FGIDs are family history [9], cow’s milk allergy [10], probable or definite anxiety [2], being an only child, initiating formula feeding at age 1 to 2 months, and a USD 11,800–23,800 annual income [11]. The Rome IV Diagnostic Questionnaire was designed and validated for the screening and diagnosis of FGIDs [12].

In Saudi Arabia, the prevalence of FGIDs among children varies according to specific disorders. A cross-sectional study in 2016 reported a 32.2% prevalence of constipation among Saudi children [9] and rates up to 7.5% for diarrhea in children under six years old [13]. A study on 4290 patients from 1995 to 2001 revealed a frequency of 2.6 per 1000 cases of cyclic vomiting syndrome [14]. Additionally, a 2015 panel reported that 32% of mothers had to change their infant formula because of infantile colic symptoms [15]. Aside from similar scattered reports, there have yet to be any nationwide studies on the matter. Nevertheless, the available data suggest that FGIDs affect a significant proportion of children living in Saudi Arabia.

The main objective of the current study was to assess the prevalence and types of FGIDs in children aged four years and older in Jeddah City and its countryside. This study also examines factors that contribute to the incidence of these disorders and their impact on children’s lifestyles. This investigation is a larger-scale follow-up to a previously published pilot study by the same research group [4], which provided the localized Arabic version of the questionnaire [16].

## 2. Materials and Methods

### 2.1. Study Design and Sample

This was a cross-sectional study conducted among 285 mothers of preschool children who were enrolled in and attended kindergartens in Jeddah City and its countryside during the academic year 2020–2021 to examine the prevalence of FGIDs.

Participants were selected using a non-random cluster sampling method. The study excluded mothers of preschool children living outside Jeddah or its countryside and whose children were younger than 3 years old.

### 2.2. Study Tool: Questionnaire

The tool used for this study was an Arabic translation of the Rome IV Diagnostic Questionnaire for the diagnosis of FGIDs in children 4 years old and over. Details on the translation process [17] the development of extra questions related to demographics and validation, and the testing of the whole survey on several populations have been previously reported [4]. The questionnaire invitation was sent through the Saudi Ministry of Education portal, which facilitated the connection with the parents.

### 2.3. Sample Size Calculation

Since this was a scaled-up investigation from the pilot study, it required the inclusion of 1600 mothers, as previously reported [4]. Briefly, based on the most recent data from the Saudi Ministry of Education for the 2019–2020 academic year, there were roughly 16,000 children enrolled in the public kindergartens in the Jeddah area. To include 1600 mothers, the minimal projected sample size was determined to be 10% of the overall population. The prevalence of GI diseases in children was estimated to be 10% based on several studies conducted in various populations [18]. Therefore, the percentage frequency of outcome factors was set at 10% ± 2 confidence limits for the sample size calculation, with a 95% confidence level and a 0.05 level of significance.

### 2.4. Statistical Analysis

The data were examined using IBM SPSS Statistics (Version 27). GraphPad Prism 9 was used to create a graph. The sociodemographic characteristics and general information about the children are presented in frequency and percentages. In order to determine the type and prevalence of the included disorders, several questions were scored using an algorithm based on the criteria provided by the Rome Foundation. The chi-square test was employed to determine the correlation between the disorders and demographic factors, as well as family history. To find predictors for FGIDs, multivariate regression analysis was used. Since the Google Forms tool requires that all questions be answered in order for the questionnaire to advance, there were no missing data. Data were considered statistically different if the *p*-value was below 0.05.

### 2.5. Ethical Approval

The research team reviewed the protocol before beginning the study and received ethical approval from the Research Ethics Committee of the Faculty of Applied Medical Science at King Abdulaziz University (Reference No.: FAMS-EC2021-03). Permission to gain access to the kindergartens was required by the School Health and Education Directorate in the Jeddah district. Consent forms were anonymously gathered from all participants, and voluntary participation was made clear before recruitment for the study.

## 3. Results

### 3.1. Demographics and Medical History of the Overall Sample

Initially, 340 responses were received. Participants whose children were below 3 years of age or who did not reside in Jeddah City or its countryside and duplicate responses were all eliminated. After elimination, the total number of included responses was 285. Approximately 54% (n = 154) of the included children were females with a mean age of 4.4 ±1.0 years old. The majority were Saudi, 88.1% (n = 251), and lived in Jeddah City, 98.2% (n = 280). Most of the children were born full term 95.4% (n = 272), and 62.1% (n = 177) were born via normal delivery (Table 1).

Seven percent (n = 20) of the surveyed children had previously been diagnosed with a GI condition. The children’s family histories were positive for several GI conditions, most commonly constipation, 36.5% (n = 104), and acid reflux, heartburn, and GERD, 35.1% (n = 100) (Table 1).

### 3.2. Type of Milk Received During the First Two Years in the Overall Sample

The majority of the children, 139 (48.8%), were fed with both breastmilk and formula; 106 (37.2%) were exclusively breastfed. The mothers were asked how long their children were breastfed. In total, 30% were breastfed for 2 to 6 months and 28% for 1.5 to 2 years. Fourteen percent were not breastfed (Table 2).

### 3.3. Patterns and Food Allergies in the Overall Sample

As shown in Table 3, two-thirds of the children, 63.9% (n = 182), were reported to have three meals a day, and 12.3% (n = 35) consumed two meals a day. Most of the children (74.7%, n = 213) had set times for meals, while 25.3% (n = 72) ate their meals at different times each day. Half of the children, 53% (n = 151), always had lunch with their families. Sixty-four percent of the mothers (n = 184) reported that their children never skip lunch during their time at kindergarten; however, one-third reported that their child skips lunch during their time at kindergarten once or twice a week. Food allergies were reported in 10.5% of the children. The most common foods causing allergy in the sample were strawberries, 2% (n = 6), and bananas, 1.7% (n = 5) (Table 3).

### 3.4. Bedtimes on Weekdays and During Weekends in the Overall Sample

Around half of the children, 48.8% (n = 139), went to bed between 9 and 11 pm on weekdays. One-third (29.5%) went to bed as late as 12 am or later. On weekends, over half of the children stayed up until midnight or later (Table 4).

### 3.5. Foods That Trigger GI Symptoms in the Overall Sample

**Figure 1:**
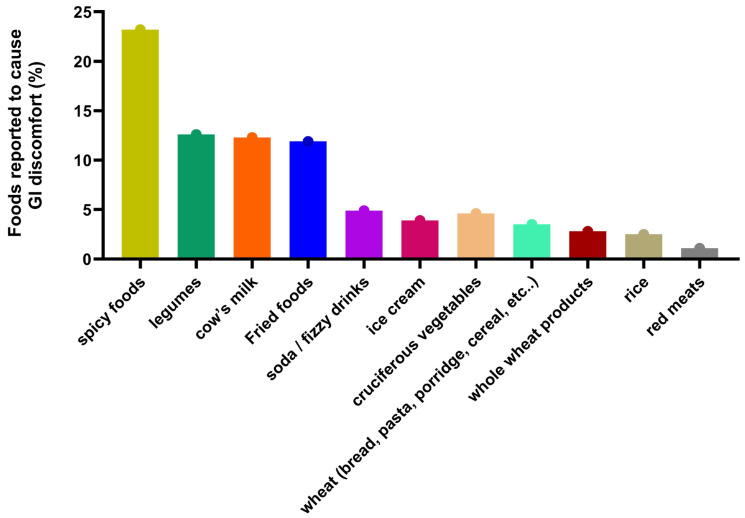
Foods reported to cause gastrointestinal discomfort when consumed by the children in the sample (N = 285).

From the various types of foods that were listed in the questionnaire, spicy foods were the most common cause of GI symptoms within the collected sample, causing symptoms in 23.2% (n = 66) of the children. Other foods causing GI discomfort included legumes, 12.6% (n = 36); cow’s milk, 11.9% (n = 34); and fried foods, 4.9% (n = 14) (Figure 1).

### 3.6. The Prevalence of FGIDs Within the Overall Sample

Of the 285 responses, 27 (9%) of the children fit the diagnostic criteria for FGIDs. The disorder with the highest prevalence was functional constipation, 3.5% (n = 10), followed by postprandial distress syndrome, 2.4% (n = 7), and functional abdominal pain—not otherwise specified, 1% (n = 3) (Table 5).

### 3.7. Association Between the Prevalence of FGID and Demographic Factors

A strong correlation was observed between children who were born preterm and the incidence of FGIDs (*p* < 0.01). There was also a remarkable association between having previously been diagnosed with a GI condition and the incidence of FGIDs in children (*p* < 0.01). Among all conditions inquired about in the family history, family history of diarrhea and family history of nausea and vomiting were significantly associated with the incidence of FGIDs (*p* < 0.001 and *p* < 0.01, respectively).

Children who consumed three meals a day were less likely to develop an FGID (*p* < 0.01). Also, a strong correlation was observed between FGID incidence and skipping lunch (*p* < 0.01) and food allergies (*p* < 0.01). A significant correlation was also observed between ice cream consumption and reported GI discomfort among children who had an FGID (*p* < 0.01) (Table 6).

### 3.8. Risk and Protective Factors for Developing FGIDs

A multivariate regression analysis was conducted to identify the most likely risk and protective factors that directly influence the development of FGIDs in children. The results showed that children born full-term (OR = 0.99, *p* < 0.01) and those who had more meals a day (OR = 0.1, *p* < 0.01) were protected from developing FGIDs. On the other hand, having parents with a family history of diarrhea was a risk factor (OR = 4.0, *p* = 0.01) (Table 7).

### 3.9. Alleviation of GI Symptoms in Participants with FGIDs

Interestingly, 88.4% of the children with FGIDs were given complementary or alternative medicine to alleviate GI symptoms: 41.9% (n = 52) were given herbal medicine; 34.4% (n = 42) were soothed with messaging/relaxation; 5.7% (n = 6) used meditation; and 1% (n = 1) used biofeedback training. None of the children were treated with acupuncture.

## 4. Discussion

The primary aim of this study was to examine the prevalence and types of FGIDs among preschool children, aged 4 to 6 years, in Jeddah and its surrounding rural areas. Moreover, this study aimed to examine factors that contribute to the incidence of these disorders and their impact on children’s lifestyles. The main outcome of the study is that 9% of the recruited sample fit the Rome IV diagnostic criteria for FGIDs. Our findings are in line with the reported prevalence of FGIDs across different regions of Saudi Arabia, which ranged from 1.6% to 41.2% [4,6,19,20,21,22]. In our study, the disorders with the highest prevalence were constipation (3.5%) and postprandial distress syndrome (2.4%), a subtype of functional dyspepsia. These results agree with previous reports on functional constipation being more prevalent in similar age groups across regions of Saudi Arabia [3,16]. In a smaller-scale study of 59 preschoolers [4], the prevalence of functional constipation was 5%, while in a larger-scale study of 317 children (ages 3–18 years), it was 4.7% [6]. Functional constipation rates in the Western Region were found to be up to 32.2% in one report [5], while 4.7% of participants met the diagnostic criteria in another [6]. These studies back up the current results and highlight how common FGIDs are in the Western Region.

### 4.1. Demographics and Medical History

Interestingly, our results showed significant associations between FGID prevalence and both gestational age and mode of delivery, in agreement with the literature. Gondim et al. found that infants born preterm had a greater chance of developing FGIDs compared with those who were full-term [23]. This can be explained by the exposure of preterm babies to factors that disrupt GI homeostasis, such as antibiotics and cesarean delivery [24]. Moreover, the mode of delivery affects the development of the gut microbiota in infants. Zhang et al. found that cesarean delivery could potentially lead to the dysbiosis of gut microbiota [12], which was found to play a role in the pathophysiology of FGIDs [13] in both adults and children [14]. Antibiotics are another factor that disrupts the gut microbiome [15]. A cohort study reported that infantile colic increased in the first year of life when antibiotics were given within the first few weeks [25,26]. Furthermore, antibiotics use in early life can lead to not only FGIDs but also food allergies [14], which, in children, have been linked to higher prevalence rates for IBS and abdominal pain syndrome [27].

The current study also showed that having a history of a GI condition was significantly associated with the occurrence of FGIDs in children. A community-based case–control study revealed that patients with a preexisting history of gastroenteritis tended to experience symptoms consistent with FGIDs such as IBS and functional diarrhea [27]. Moreover, the current study showed that the risk of having an FGID increased in children who had a positive family history of nausea and vomiting or diarrhea, in agreement with similar reports in the literature [4,5,9]. This could be explained by a 2009 microarray analysis that found that family members share gut microbiomes through levels of shared genes [28], meaning that if parents have an altered gut microbiota, their child might inherit it and, thus, have a higher risk of acquiring FGIDs.

### 4.2. Eating Patterns and Food Allergies

Twenty-five percent of the children surveyed did not have their meals at set times, and the data analysis revealed a correlation between irregular meal patterns and an increased risk of FGIDs. Furthermore, one-third to half of the children stayed up until midnight or later. According to reports, late-night eating impairs the gut barrier function through decreased mucus secretion, elevated infiltration of inflammatory cells, and altered gut microbiota composition and diversity, all of which can contribute to the pathophysiology of FGIDs [27]. The same study found that a higher score on the IBS-Symptom Severity Scale (IBS-SSS) was associated with irregular dietary habits in adults [7]. Additionally, a cross-sectional study conducted on children living in the Eastern Region found that children regularly consumed low-fiber foods such as chips, pizza, and biscuits [8]. It is well-known that poor diet quality is associated with functional constipation during childhood [9,10]. This highlights the major role diet can play in the prevalence of functional constipation among children in Saudi Arabia and the importance of following a healthy lifestyle from the early years of life. Other previously hypothesized reasons for the high prevalence of functional constipation include being uncomfortable with using the school lavatory to pass stools, not getting adequate hours of sleep, and/or having time-consuming homework [29]. Furthermore, Olaru et al. found that the prevalence of functional constipation was higher in children raised by a single parent, who live in broken families, or who come from orphanages [9,11]. This should draw attention for the purposes of public health campaigns to shed light on the dietary, lifestyle, and psychological needs of children to help manage the symptoms they experience.

Haapalahti et al. found that children with FGIDs tend to skip lunch at school more often and experience feelings of anxiety and discomfort. They assessed food habits in children with FGIDs and found that most of the children had irregular dietary patterns as a result of the family’s lack of cooperation with the child to provide regular meals. In order to manage and prevent the severity of FGID symptoms, it is crucial that families are educated on how to deal with these disorders [30].

In the current study, food allergies were found to have a strong association with FGIDs. This finding aligns with the results of a cohort study by Kamphorst et al., who found that children with parent-reported food allergies were more likely to have FGIDs [27]. The increased risk for FGIDs due to food allergies may have several explanations. The increased risk may be a direct effect of allergies through immunoglobulin E and a mast-cell-dependent mechanism after the ingestion of dietary antigens [31]. Another explanation is that allergies and FGIDs have similar pathophysiological mechanisms; for instance, inflammatory signals and increased gut permeability are found in both conditions [28,32]. Thus, healthcare professionals should be cautious when dealing with children under 2 years of age, as some medical interventions could lead to detrimental long-term effects.

### 4.3. Foods Causing Discomfort and Symptom Alleviation

Different foods have been reported to have a positive association with FGIDs. Suggested mechanisms for the discomfort induced by food in people with FGIDs include abnormal gas handling, malabsorption, abnormal colonic fermentation, food allergies, visceral hypersensitivity, altered secretion, psychological factors, activation of motor responses in the GI tract, and the stimulation of chemoreceptors or mechanoreceptors [33,34]. Altered motility, especially, leads to unpleasant GI symptoms with the consumption of high-fat foods such as ice cream and full-fat cow’s milk [35,36]. Besides impacting children’s health, FGIDs impact their quality of life. Pain-inducing FGIDs were seen to decrease quality of life and increase school absence [37].

Interestingly, approximately 88.4% of the parents in the current study used different types of complementary and alternative medicine when dealing with FGID symptoms. Parents turn to complementary or alternative medicine for reasons such as pain relief, dissatisfaction with conventional medicine, fear of using more medication, and simply preferring an overall more natural remedy for their children, as reported in a multicenter survey on alternative medicine use among pediatric patients [31,37]. There have been reports on the positive effect of some herbs, such as peppermint, on FGID symptoms through the inhibition of smooth muscle contraction [38,39]. Moreover, meditation was found to help reduce anxiety and symptoms in FGIDs such as IBS. Also, acupuncture, a physical approach, restores the health of an organ by restoring the flow of qi (energy that circulates among organs through meridian channels) [34,36,40].

### 4.4. Strength and Limitation

A major strength of this study is the use of the self-reported Rome IV Questionnaire, which allows for diagnosis without the need for invasive procedures or physical assessment. This approach was extremely beneficial since the study was conducted during the COVID-19 pandemic when physical contact with children or their parents was not possible. Moreover, the questionnaire has been used and validated by multiple researchers and clinicians in the past [41,42,43]. The translated questionnaire is this study’s core strength. This is the first translated version available in Arabic specific to Saudi Arabia, offering researchers and clinicians in the region a diagnostic tool for assessing FGIDs. Another strength is that the study tool was distributed online in cooperation with the Saudi Ministry of Education, which contributed to a wider distribution to reach the broadest number of mothers possible.

The greatest limitation, however, was not reaching the required sample size. Thus, the findings should be interpreted as an approximate indicator of FGID prevalence among preschool children. In spite of the limited number of studies conducted during the pandemic on the same age group, the same challenge was seen to affect other studies on FGID prevalence [44]. This can further be attributed to factors such as reduced access to participants, given the constraints set by the Saudi Ministry of Education, and the limited access to the intended population, although the same precautions did not prevent preschool children from attending kindergarten. For future studies, we recommend having face-to-face meetings with parents to fill out the questionnaire and gather objective anthropometric data from the children to associate them with FGID prevalence. It would also be interesting if future studies examined the relationship between FGIDs and gut–brain interactions, which would explore the role of the microbiota and advance our understanding of complex pathophysiology. Nevertheless, the results from this study can be used to compare different regions in Saudi Arabia. Our study’s reliance on self-reported data at a single time point and anonymous collection rather than conventional methods to objectively verify data can be viewed as a limitation. However, addressing these weaknesses was not feasible given the challenges of conducting such research during the COVID-19 national lockdown.

## 5. Conclusions

FGIDs are prevalent among 9% of children in Jeddah and its countryside. The most common FGID is functional constipation. Factors associated with FGIDs in children include preterm birth, being previously diagnosed with a GI condition, irregular eating habits, food allergies, and having a positive family history of GI conditions. The findings of this study are expected to ignite further, larger studies that will hopefully lead to determining the prevalence of FGIDs at a national level.

## Figures and Tables

**Table 1:** Demographic features and medical history of the overall sample (N = 285).

Variable			Mean ± SD or n (%)	
Age	4.4 ± 1.0 years			
Gender	Female		154 (54.0%)	
	Male		131 (46.0%)	
Nationality	Non-Saudi		34 (11.9)	
	Saudi		251 (88.1%)	
Residence	Countryside of Jeddah		5 (1.8%)	
	Jeddah City		280 (98.2)	
Medical history				
Type of delivery	Cesarean section		108 (37.9)	
	Normal		177 (62.1%)	
Gestational age at birth	Full term		272 (95.4%)	
	Preterm		13 (4.6%)	
Any previously diagnosed GI conditions?	Yes		20 (7.0%)	
	No		256 (93.0%)	
Family history				
Dyspepsia/indigestion	Yes		36 (12.6%)	
	No		249 (87.3%)	
Acid reflux, heartburn, GERD	Yes		92 (32.2%)	
	No		193 (67.7)	
Nausea and vomiting	Yes		28 (9.8%)	
	No		257 (90.1%)	
Peptic ulcer disease	Yes		17 (5.9%)	
	No		268 (94%)	
Abdominal pain syndrome	Yes		14 (4.9%)	
	No		271 (95.08%)	
Flatulence, bloating, belching	Yes		62 (21.7%)	
	No		223 (78.2%)	
Pancreatitis, gallstone and gallbladder disorders, biliary tract disorders	Yes		21 (7.3%)	
	No		264 (92.6%)	
Constipation	Yes		94 (32.9%)	
	No		191 (67.01%)	
Diarrhea	Yes		34 (11.9%)	
	No		251 (88.07%)	
Crohn’s disease	Yes		6 (2.1%)	
	No		279 (97.8%)	
Total	(N = 285)			

SD = standard deviation; n = sample of participants; GI = gastrointestinal; GERD = gastroesophageal reflux disease.

**Table 2:** Type of milk and duration of breastfeeding during the first two years of the overall sample (N = 285).

	No.	%
Type of feeding		
Breastfed exclusively	106	37.2
Formula-fed exclusively	40	14
Both breast milk and formula	139	48.8
Duration of breastfeeding		
One month	30	11%
2–6 months	87	30%
7 months–1 year	49	17%
1.5–2 years	79	28%
Was not breastfed	40	14%

**Table 3:** Eating patterns and food allergies among the overall sample (N = 285).

	No.	%
Number of meals per day		
1	3	1.1
2	35	12.3
3	182	63.9
4	65	22.8
Mealtimes are set		
Yes	213	74.7
No	72	25.3
Does the child have lunch with the family?		
Always	151	53
Sometimes	130	45.5
No	4	1.4
Skipping lunch during their time at kindergarten		
Never	184	64
Once a week	50	17.5
Twice a week	41	14.4
3 times a week	9	3.2
4 times a week	1	0.4
Food allergies		
Strawberry	6	2
Banana	5	1.7
Honey	3	1
Almond	2	0.7
Fish	2	0.7
Chocolate	1	0.3
Mango	1	0.3
Seafood	1	0.3
Nuts	1	0.3
Wheat	1	0.3
G6PD	1	0.3
Cream cheese	1	0.3
Dairy	2	0.7
Oranges	2	0.7

**Table 4:** Reported bedtimes for the overall sample (N = 285).

	No.	%
Bedtime on weekdays		
Before 6 pm	2	0.7
6–8 pm	60	21.1
9–11 pm	139	48.8
12 am or later	84	29.5
Bedtime during weekends		
Before 6 pm	4	1.4%
6–8 pm	18	6.3%
9–11 pm	106	37.2%
12 am or later	157	55.1%

**Table 5:** Functional gastrointestinal disorders diagnosed within the sample (n = 27).

FGID	Number of Children That Met the Diagnostic Criteria	Percentage (%)
Functional constipation	10	3.50%
IBS	1	0.30%
Postprandial distress syndrome	7	2.40%
Functional abdominal pain—NOS	3	1%
Functional epigastric pain	2	0.70%
Cyclic vomiting syndrome	2	0.70%
Aerophagia	1	0.30%
Non-retentive fecal incontinence	1	0.30%

FGID = functional gastrointestinal disorder; IBS = irritable bowel syndrome; NOS = not otherwise specified.

**Table 6:** Association between the prevalence of FGIDs and demographic factors.

Demographic Factors			FGIDs n (%)		Total n (%)	*p*-Value
			No FGID (n = 258)	FGID (n = 27)	(n = 285)	
**Gestational age at birth**		Full term	252 (97.29%)	20 (76.92%)	272 (95.43%)	<0.01 *
		Preterm	7 (2.70%)	6 (23.07%)	13 (4.56%)	
Previously diagnosed GI condition		Positive	15 (5.79%)	5 (19.23)	20 (7.01)	<0.01 *
Family history of GI conditions	diarrhea	Positive	31 (11.96%)	10 (38.46%)	41 (14.38%)	<0.001 ***
	nausea and vomiting	Positive	29 (11.19%)	8 (30.76%)	37 (12.98%)	<0.01 **
Number of meals per day		1.00	1 (0.38%)	2 (7.69%)	3 (1.05%)	<0.01 **
		2.00	26 (10.03%)	9 (34.61%)	35 (12.28%)	
		3.00	173 (66.79%)	9 (34.61%)	182 (63.85%)	
		4.00	59 (22.77%)	6 (23.07%)	65 (22.80%)	
Number of times skipping lunch during their time at kindergarten		0.00	170 (65.637%)	14 (53.84%)	184 (64.56%)	<0.01 **
		1.00	43 (16.60%)	7 (26.92%)	50 (17.54%)	
		2.00	37 (14.28%)	4 (15.38%)	41 (14.38%)	
		3.00	9 (3.47%)	0 (0%)	9 (3.15%)	
		4.00	0 (0%)	1 (3.84%)	1 (0.35%)	
Food allergy		Positive	22 (8.49%)	8 (30.76%)	30 (10.52%)	<0.01 **
Foods reported to cause gastrointestinal discomfort	Fried foods	Positive	28 (10.81%)	6 (23.07%)	34 (11.92%)	<0.01 **
	Spicy foods	Positive	60 (23.16%)	6 (23.07%)	66 (23.15%)	>0.05
	Cow’s milk	Positive	26 (10.03%)	9 (34.61%)	35 (12.28%)	<0.01 **
	Rice	Positive	4 (1.54%)	3 (11.53%)	7 (2.45%)	<0.05 *
	Wheat (bread, pasta, porridge, cereal, etc.)	Positive	8 (3.08%)	2 (7.69%)	10 (3.50%)	>0.05
	Whole wheat products	Positive	8 (3.08%)	0 (0%)	8 (2.80%)	>0.05
	Cruciferous vegetables	Positive	12 (4.63%)	1 (3.84%)	13 (4.56%)	>0.05
	Legumes	Positive	34 (13.12%)	2 (7.69%)	36 (12.63%)	>0.05
	Soda/fizzy drinks	Positive	14 (5.40%)	0 (0%)	14 (4.91%)	>0.05
	Ice cream	Positive	7 (2.70%)	4 (15.38%)	11 (3.85%)	<0.01 **
	Red meats	Positive	3 (1.15%)	0 (0%)	3 (1.05%)	>0.05

Chi-square test was used. Abbreviations: FGIDs, functional gastrointestinal diseases. * *p* < 0.05, ** *p* < 0.01, and *** *p* < 0.001.

**Table 7:** Risk and protective factors for developing FGIDs (N = 285).

Factor	*p*-Value	OR	95% C.I. for OR	
			Lower	Upper
Full term	<0.01 **	0.099	0.028	0.354
Number of meals consumed per day	<0.01 **	0.100	0.012	0.815
Family history of diarrhea	0.012 *	4.064	1.362	12.124

Multinomial regression analysis was used. Abbreviations: FGIDs, functional gastrointestinal diseases; OR: odds ratio; CI: confidence interval. * *p* < 0.05 and ** *p* < 0.01.

## Data Availability

All new data were published along this article.
